# Machine learning for predicting *antimicrobial efficacy* of periodontal gel formulations *in vitro* biofilm models

**DOI:** 10.6026/973206300213866

**Published:** 2025-10-31

**Authors:** Rohitkumar R Thakkar, Nirma Yadav, Anand Kumar, Shilpa Duseja, Sunny Mavi, Udipta Sahoo

**Affiliations:** 1 Department of Periodontology, Siddhpur Dental College and Hospital, Siddhpur - 384151, Gujarat, India; 2 Department of Dentistry, Maa Vindhyawasini Autonomous State Medical College, Mirzapur, Uttar Pradesh, India; 3 Department of Dentistry, Moti Lal Nehru Medical College, Prayagraj, Uttar Pradesh, India; 4 Department of Periodontology, Narsinhbhai Patel Dental College and Hospital, Sankalchand Patel University, Visnagar, India; 5 Department of Periodontics, Sudha Rustagi College of Dental Sciences and Research, Faridabad, Haryana, India; 6 Department of Periodontics, Kalinga Institute of Dental Sciences, KIIT (Deemed to be university), Patia, Bhubaneswar, Odisha, India

**Keywords:** machine learning, periodontal gel, *antimicrobial efficacy*, biofilm, prediction model, drug formulation, chlorhexidine, chitosan

## Abstract

Periodontal disease caused by dysbiotic biofilms poses a major challenge and predicting the efficacy of topical antimicrobial gels is limited by biofilm resistance and resource-intensive *in vitro* testing. Therefore, it is of interest to develop machine learning (ML) models to predict *antimicrobial efficacy* of novel gel formulations against multi-species periodontal biofilms. Hence, a total of 120 formulations with varying polymers, agents, concentrations and enhancers were tested using the Calgary Biofilm Device and efficacy data were used to train Random Forest, SVM, Gradient Boosting and Neural Network models. Gradient Boosting achieved the best performance (accuracy 92.8%, AUC-ROC 0.96), with antimicrobial type, concentration and polymer *viscosity* as key predictors. ML, particularly Gradient Boosting, offers a reliable tool for predicting periodontal gel efficacy, enabling faster formulation optimization and reducing the need for extensive laboratory screening.

## Background:

Periodontitis, a chronic inflammatory disease affecting nearly 50% of adults globally, is primarily initiated and sustained by pathogenic biofilms accumulating on tooth surfaces and within periodontal pockets [1]. These biofilms, characterized by complex polymicrobial communities encased in an extracellular polymeric substance (EPS), exhibit significantly enhanced resistance to antimicrobial agents compared to their planktonic counterparts [2]. Mechanical debridement (scaling and root planing) remains the cornerstone of treatment; however, its limitations in accessing deep pockets and complex root anatomy necessitate adjunctive antimicrobial therapies [3]. Topical antimicrobial gels offer a promising strategy by delivering high local concentrations of agents directly to the periodontal pocket, minimizing systemic side effects and improving patient compliance [4]. Despite their potential, the clinical efficacy of existing topical antimicrobials is often variable and suboptimal. This variability stems from multiple factors, including the inherent resistance of biofilms, limited penetration of antimicrobials through the EPS and into deeper layers of the biofilm and the physicochemical properties of the gel vehicle itself influencing drug release kinetics and substantivity [5]. Developing novel, highly effective gel formulations requires extensive and costly iterative *in vitro* and *in vivo* testing against representative biofilm models. Traditional *in vitro* biofilm assays, while essential, are laborious, time-consuming (often taking 5-7 days per formulation) and resource-intensive, creating a significant bottleneck in the drug development pipeline [6]. Recent advances in computational modeling and artificial intelligence present transformative opportunities for pharmaceutical research. Machine learning (ML) algorithms excel at identifying complex, non-linear relationships within high-dimensional datasets, making them ideally suited for predicting biological outcomes based on compound or formulation characteristics [7]. ML has been successfully applied in various biomedical contexts, including predicting drug-target interactions, optimizing drug delivery systems and even identifying novel antimicrobial peptides [8]. Within dentistry, ML models have shown promise in diagnosing periodontal disease from radiographs and predicting treatment outcomes [9]. However, the application of ML specifically for predicting the *antimicrobial efficacy* of topical periodontal formulations against complex, multi-species biofilms remains largely unexplored [10]. A critical research gap exists in leveraging computational approaches to rapidly screen and optimize periodontal gel formulations. Current development relies heavily on empirical testing, lacking efficient predictive tools that could prioritize the most promising candidates for experimental validation. Furthermore, understanding the relative contribution of different formulation components (*polymer type*, antimicrobial agent, concentration, enhancers) to overall biofilm efficacy through data-driven modeling is lacking [11]. Therefore, it is of interest to develop, train and validate machine learning models capable of accurately predicting the *antimicrobial efficacy* of diverse periodontal gel formulations against a validated multi-species *in vitro* biofilm model, based solely on their physicochemical and compositional properties.

## Materials and Methods:

## Study design:

An experimental *in vitro* study combined with computational modeling.

## Sample size:

120 unique gel formulations were synthesized. Each formulation was tested in quintuplicate (n=5 replicates) against biofilms, yielding 600 data points.

## Formulation design and inclusion criteria:

[1] Polymer Base: Carbopol 940 (0.5-2.0% w/v), Chitosan (low, medium, high molecular weight; 1.0-2.5% w/v), Poloxamer 407 (18-25% w/v).

[2] Antimicrobial Agent: Chlorhexidine digluconate (CHX; 0.1-2.0% w/v), Doxycycline hyclate (DOX; 0.5-2.0% w/v), Cetylpyridinium chloride (CPC; 0.05-0.2% w/v).

[3] Penetration Enhancer: None, Disodium EDTA (0.5-1.0% w/v), Citric Acid (0.5-1.0% w/v).

[4] Other Components: Glycerol (humectant, 5-10% w/v), Methylparaben (preservative, 0.1% w/v), Deionized water (q.s. to 100%).

[5] Inclusion: Formulations with pH 5.5-7.5, *viscosity* 5,000-50,000 cP (measured by Brookfield DV2T viscometer) and acceptable physical stability (no phase separation/syneresis after 1 week at 4°C, 25°C, 40°C).

[6] Exclusion: Formulations exhibiting precipitation, instability, or *viscosity* outside the target range.

## Biofilm model:

[1] Bacterial Strains: *Streptococcus gordonii* ATCC 10558, *Fusobacterium nucleatum* ATCC 25586, *Porphyromonas gingivalis* ATCC 33277.

[2] Culture Conditions: Strains maintained anaerobically (80% N_2_, 10% H_2_, 10% CO_2_) at 37°C in Brain Heart Infusion (BHI) broth supplemented with hemin (5 µg/mL) and menadione (1 µg/mL).

[3] Biofilm Growth: Biofilms grown on pegs of Calgary Biofilm Devices (CBD, Innovotech Inc.) for 72 hours anaerobically at 37°C. Medium was refreshed at 24h and 48h. Mature biofilms were confirmed by scanning electron microscopy (SEM) on representative pegs.

## *antimicrobial efficacy* testing:

[1] Treatment: Biofilm-coated pegs were exposed to 200 µL of each gel formulation for 60 minutes anaerobically at 37°C. Controls included untreated biofilms (negative) and 0.2% CHX solution (positive).

[2] Recovery and Quantification: Treated pegs were rinsed twice in PBS, sonicated (40 kHz, 5 min) in 1 mL PBS to dislodge biofilm and vortexed (30 sec). Serial dilutions were plated on BHI blood agar plates. Plates were incubated anaerobically for 5-7 days and viable colonies (CFU/mL) were counted.

[3] Efficacy Metric: Log_10_ reduction = Log_10_(CFU/mL control) - Log_10_(CFU/mL treated). Formulations were categorized as "High Efficacy" (≥3 log_10_ reduction) or "Low Efficacy" (<3 log_10_ reduction) for classification tasks.

## Machine learning pipeline:

[1] Data splitting: Dataset randomly split into training (70%, n=420) and testing (30%, n=180) sets, stratified by efficacy category.

[2] Preprocessing: Categorical features one-hot encoded. Numerical features standardized (z-score normalization).

[3] Model selection and training: Four algorithms were implemented using Python (scikit-learn, TensorFlow):

1) Random Forest (RF)

2) Support Vector Machine (SVM; RBF kernel)

3) Gradient Boosting (GB; XGBoost implementation)

4) Neural Netswork (NN; 3 hidden layers [64, 32, 16 neurons], ReLU activation, Adam optimizer) Models trained on the training set with 5-fold cross-validation for hyperparameter tuning (e.g., n_estimators, max_depth for RF/GB; C, gamma for SVM; epochs, batch size for NN).

[4] Performance evaluation (Testing Set):

1) Classification (High/Low Efficacy): Accuracy, Precision, Recall, F1-Score, AUC-ROC.

2) Regression (Log_10_ Reduction): Mean Absolute Error (MAE), Root Mean Squared Error (RMSE), R^2^.

[5] Feature Importance: Computed using permutation importance (RF, GB, SVM) and SHAP (SHapley Additive exPlanations) values for GB.

[6] Baseline Comparison: Multiple Linear Regression (MLR) was trained and evaluated for the regression task.

## Statistical analysis:

Experimental data presented as mean ± standard deviation (SD). Differences in log_10_ reduction between formulation groups assessed by one-way ANOVA with Tukey's post-hoc test. Differences in ML model performance metrics assessed by paired t-tests (Bonferroni-corrected for multiple comparisons). Significance level was p < 0.05 (SPSS v28.0, IBM; Python SciPy/Statsmodels).

## Results:

Significant differences in *antimicrobial efficacy* were observed across formulation types (p < 0.001). Chitosan-based gels generally outperformed Carbopol and Poloxamer. The highest efficacy was achieved by Chitosan (High MW) + 1.5% CHX + 1.0% EDTA (4.2 ± 0.3 log_10_ reduction). Poloxamer gels with CPC showed the lowest efficacy (1.8 ± 0.4 log_10_ reduction). All ML models significantly outperformed random guessing (AUC-ROC > 0.5, p < 0.001). Gradient Boosting (GB) demonstrated superior overall performance:

Gradient Boosting (GB) demonstrated superior overall performance:

[1] Accuracy: GB (92.8 ± 2.1%) > RF (89.5 ± 2.4%) > NN (87.3 ± 3.0%) > SVM (85.1 ± 2.8%)

[2] AUC-ROC: GB (0.96 ± 0.03) > RF (0.93 ± 0.04) > NN (0.91 ± 0.05) > SVM (0.89 ± 0.04) GB showed significantly higher accuracy and AUC-ROC than SVM and NN (p < 0.01) and higher AUC-ROC than RF (p < 0.05).

GB again achieved the best predictive performance for the continuous efficacy metric:

[1] MAE: GB (0.32 ± 0.08) < RF (0.41 ± 0.09) < NN (0.48 ± 0.11) < SVM (0.55 ± 0.12)

[2] R^2^: GB (0.88) > RF (0.82) > NN (0.78) > SVM (0.73) GB's MAE was significantly lower than all other models (p < 0.01) and its R^2^ was significantly higher (p < 0.01). GB significantly outperformed the MLR baseline (MAE: 0.81 ± 0.12, R^2^: 0.61; p < 0.001) (Table 1-3 - see PDF).

Permutation importance and SHAP analysis identified the most influential features:

[1] Antimicrobial Agent Type (Importance: 24.7%) - CHX > DOX > CPC

[2] Antimicrobial Concentration (18.9%) - Positive correlation with efficacy

[3] Polymer *viscosity* (15.3%) - Moderate *viscosity* (~20,000 cP) optimal

[4] *polymer type* (12.8%) - Chitosan > Carbopol > Poloxamer

[5] Enhancer Type (11.2%) - EDTA > Citric Acid > None

[6] Enhancer Concentration (8.1%) - Positive correlation

[7] pH (5.4%) - Near-neutral pH optimal Other features showed minor contributions (<5%).

## Discussion:

This study demonstrates the successful development and validation of machine learning models, particularly Gradient Boosting, for accurately predicting the *antimicrobial efficacy* of periodontal gel formulations against a clinically relevant multi-species biofilm model. The high predictive accuracy (92.8% classification, MAE 0.32 log_10_ reduction) achieved by GB highlights the significant potential of ML to streamline the development of novel topical antimicrobials for periodontitis management. The superior performance of GB aligns with its known strength in handling heterogeneous tabular data with complex interactions between features, which is characteristic of pharmaceutical formulation datasets [12]. Its ability to capture non-linear relationships likely explains its significant advantage over traditional linear regression, which failed to model the intricate dependencies between formulation components and biofilm killing effectively. The relatively strong performance of RF and NN further supports the utility of ensemble and deep learning approaches for this task, while SVM, while competent, was less optimal, possibly due to sensitivity to feature scaling or kernel choice [13]. The feature importance analysis provides crucial, data-driven insights into formulation design. The dominant influence of the antimicrobial agent type and its concentration was expected, as CHX and DOX are well-established broad-spectrum antimicrobials effective against periodontal pathogens, while CPC is generally considered less potent against anaerobes like *P. gingivalis* [14]. The strong positive correlation between concentration and efficacy is consistent with dose-response relationships. However, the significant contribution of *polymer type* and *viscosity* underscores the critical, often underappreciated, role of the vehicle. Chitosan's prominence aligns with its inherent antimicrobial and biofilm-disrupting properties, likely synergizing with the loaded antimicrobial agent [15]. The optimal *viscosity* range identified (~20,000 cP) suggests a balance between sufficient biofilm contact/adhesion and adequate diffusion/release kinetics - too low *viscosity* may reduce retention, while too high may impede drug release [16]. The positive impact of *penetration enhancers*, particularly EDTA, is mechanistically plausible. EDTA chelates divalent cations (Ca^2+^, Mg^2+^) essential for EPS integrity and membrane stability, disrupting the biofilm barrier and enhancing antimicrobial penetration [17]. Citric acid, while also chelating, may be less effective at neutral pH or could cause local irritation. The finding that near-neutral pH is optimal likely reflects the stability of the antimicrobials (especially CHX) and the physiological pH of the periodontal pocket [18]. The experimental results validating the top-performing formulations (e.g., Chitosan + 1.5% CHX + EDTA achieving >4 log_10_ reduction) are clinically significant, exceeding the typical efficacy of commercially available CHX gels (often ~2-3 log_10_ reduction in similar models) [19]. This demonstrates the potential of rationally designed formulations guided by ML predictions. The lower efficacy of Poloxamer-based gels, particularly with CPC, might be attributed to the thermosensitive nature of Poloxamer (potentially reducing substantivity at body temperature) and CPC's known susceptibility to inactivation by divalent cations and organic matter prevalent in biofilms [20].

## Conclusion:

Machine learning, particularly Gradient Boosting, provides a powerful and accurate tool for predicting periodontal gel efficacy against complex biofilms. By identifying key formulation factors such as antimicrobial type, concentration, polymer base and enhancers, it enables rational design of superior therapies. This computational approach can accelerate innovation while reducing reliance on resource-intensive empirical screening for periodontitis management.

## References

[1] Ebrahimi Tarki F (2023). Iran J Pharm Res..

[2] Bose B (2022). Front Microbiol..

[3] Sukmarini L (2024). J Nat Med..

[4] Abdelhamid AG (2023). Antibiotics (Basel)..

[5] Li Y (2022). Microbiol Spectr..

[6] Kumar S (2023). Comput Struct Biotechnol J..

[7] Cao Y (2020). Amino Acids..

[8] Hwang HJ (2021). Microbiol Spectr..

[9] Boone K (2021). BMC Bioinformatics..

[10] Haney EF (2018). Biomolecules..

[11] LoVetri K (2010). Methods Mol Biol..

[12] Teerapo K (2019). PLoS One..

[13] León Madrazo A (2022). Comput Biol Chem..

[14] Boone K (2018). BMC Bioinformatics..

[15] Wei M (2023). Front Cell Infect Microbiol..

[16] Arif M (2024). ACS Omega..

[17] Manobala T. (2025). Crit Rev Microbiol..

[18] Shaban TF (2022). Comput Biol Med..

[19] Chatupheeraphat C (2023). Front Cell Infect Microbiol..

[20] Alhhazmi AA (2024). Microbiol Spectr..

